# Long noncoding RNA UCA1 regulates HCV replication and antiviral response via miR-145-5p/SOCS7/IFN pathway

**DOI:** 10.7150/ijbs.59227

**Published:** 2021-07-05

**Authors:** Haiyan Zeng, Lei Li, Yi Gao, Guojun Wu, Zhouhua Hou, Shuiping Liu

**Affiliations:** 1 Department of Microbiology, School of Basic Medical Science, Central South University, Changsha, Hunan 410078, China.; 2 Department of Laboratory Medicine, Peking Union Medical College Hospital, Peking Union Medical College & Chinese Academy of Medical Science, Beijing 100010, China.; 3 Department of Infectious Disease, the Hainan Affiliated Hospital of Hainan Medical University, Haikou, Hainan 570311, China.; 4 Department of Infectious Disease, Xiangya Hospital, Central South University, Changsha, Hunan 410008, China.

**Keywords:** Hepatitis C virus, urothelial carcinoma-associated 1, microRNA, suppressor of cytokine signaling 7, antiviral response

## Abstract

Hepatitis C virus (HCV) infection involves a variety of viral and host factors, which leads to the dysregulation of number of relevant genes including long noncoding RNAs (LncRNAs). LncRNA urothelial carcinoma-associated 1 (UCA1) has been reported to be upregulated in HCV-infected individuals. In a bid to elucidate on the contribution of UCA1 on HCV replication, we infected Huh7.5 cells with cell culture-derived HCV and found that UCA1 expression was elevated in time- and dose-dependent manners. Functionally, UCA1 knockdown by siRNA upregulated interferon (IFN) responses, thereby increasing the expression of interferon-stimulating genes (ISGs), and subsequently suppressing HCV replication. Bioinformatics analysis and experimental results indicated that, functioning as competitive endogenous RNA, UCA1 could sponge microRNA (miR)-145-5p, which targeted suppressor of cytokine signaling 7 (SOCS7) mRNA and subsequently mediated SOCS7 silencing. Moreover, SOCS7 protein exerted an inhibitory effect on IFN responses, thereby facilitating HCV replication. Taken together, at first, our findings demonstrate that UCA1 can counteract the expression of miR-145-5p, thereby upregulating the level of SOCS7, and in turn leading to the suppression of antiviral response in Huh7.5 cells.

## Introduction

Hepatitis C virus (HCV) is a hepatotropic non-cytopathic positive strand RNA virus, which is considered as the main initiator of chronic hepatitis and hepatic metabolic disorders. These disorders may eventually deteriorate to cirrhosis and hepatocellular carcinoma (HCC) 1. Although effective anti-HCV drugs such as IFN alpha (IFN-α), ribavirin and direct acting antivirals (DAAs) have been developed and put into clinical use for some time now, the insufficient preventive approaches and the recessive development result in a large number of patients diagnosed at late stage of HCV infection as progressed liver disorders or even HCC 2, 3. Until today, with the growing cases of incidence, HCC is a major health problem worldwide. Despite improvements in diagnosis and treatment of HCC, the rate of lives saved remains low 4. In response to this emergency, mechanistic studies underlying host-virus interaction are critical for improving the survival rate of patients with HCV infection.

In the recent decade, long non-coding RNAs (lncRNAs) are known as a new class of transcripts, which are universally discovered in the mammalian genome. They function as the critical regulators of the many cellular processes, including but not limited to tumor growth and development, apoptosis, proliferation, differentiation, and cell autophagy 5. Increasing evidence has revealed that aberrant lncRNA expression is associated with many human diseases such as liver fibrosis and HCC 6. For instance, in a study of Yuan* et al*, they found that lncRNA-ATB acts as competing endogenous RNAs (ceRNAs) through competitively sponging the miR-200 family to suppress epithelial-mesenchymal transition (EMT), invasion, and metastasis of HCC 7. Moreover, lncRNA Myd88 promotes the growth and metastasis of HCC through regulating Myd88 expression via H3K27 modification 8. Additionally, more lncRNAs such as HULC, MVIH, HOTAIR and the likes have been found to play roles in HCC 6, 9, 10.

The lncRNA urothelial carcinoma-associated 1 (UCA1), derived from chromosome 19p13.12, was found to be upregulated in bladder tumor and proved to be an oncogene in many cancers 11, 12. Number of studies have shown that lncRNA UCA1 can modulate the Wnt pathway through interacting with DNA, mRNA, microRNA (miRNA) and protein, thus affecting the biological activities of tumors 13. It has been shown that expression of lncRNA UCA1 was upregulated in the sera and tissue of HCC patients and relevant to Child-Pugh score 13, 14. However, the functions and the underlying mechanisms of UCA1 in HCV infection have not yet been investigated. A major limitation from previous studies is that lncRNAs were analyzed in HCC regardless of the hepatitis virus involved. A general contrasting phenomena to this is that, only a few virus-regulated lncRNAs have been identified and moreover, these functions has been poorly studied.

microRNA-145-5p (miR-145-5p) has been frequently reported as a tumor suppressor. A good number of studies have shown that the expression of miR-145-5p is down-regulated in a variety of human tumors, such as colon cancer 15, bladder cancer 16 and as well as prostate cancer 17. Moreover, miR-145-5p can target multiple molecules in cells, including c-myc 18, fascin1 19, yes 20, ilk 21 and klf4 22, to exert its effect. In addition, it is known that miR-145-5p can inhibit tumor development by regulating cell growth, invasion and metastasis 23, 24. In response to the mentioned contribution of miR-145-5p, it is likely to play a pivotal role in the antiviral response of HCC.

Recently, strong evidence has been established on the expression of the suppressor of cytokine signaling (SOCS) protein family (SOCS1-SOCS7 and cytokine-inducible SH2-containing protein (CIS)) under cytokine stimulation, and it can inhibit a variety of cytokine signaling pathways in cells as a suppressor 24, 25.

Generally, the regulatory relationship between a specific cytokine and the induced SOCS mRNA is not constant. Indeed, the expression levels of SOCS family members induced by cytokines tend to vary among the specific cell lines or tissues 25. For example, interleukin (IL)-10 and lipopolysaccharide can specifically stimulate SOCS3 expression in monocytes and macrophages, respectively 26, 27. Interestingly, in a study of Diehl *et al*, they found that IL-6 could increase SOCS1 expression in CD4^+^ T cells and then suppress IFN-γ signaling, thereby restraining the differentiation of CD4^+^ Th1 cell 28. However, limited number of studies focused on the function of SOCS7 in the development of HCC and HCV replication.

In this study, we aimed to investigate whether the expression of UCA1 is associated with HCV replication and its underlying mechanism. The result suggests that UCA1 is conducive to HCV replication in Huh7.5 cells by targeting miR-145-5p to upregulate SOCS7 expression, and ultimately inhibiting the antiviral response.

## Materials and Methods

### Cell culture

Human hepatocarcinoma derived cell line Huh7.5 (Laboratory of Molecular Virology, Fudan University) was incubated in Dulbecco's modified Eagle's medium (DMEM) (Invitrogen) supplemented with 10% fetal bovine serum (FBS) (Gibco), 1% penicillin (10,000 U/mL)-streptavidin (10,000 μg/mL), and grown at 37 °C in a 5% CO_2_ incubator.

### Cell transfection

The miR-145-5p (mimics), miR-145-5p inhibitor, UCA1 siRNAs, SOCS7 siRNAs, and the corresponding negative controls were synthesized by Ruibo Biotechnology (Guangzhou, China). The plasmid pCDNA3.1-SOCS7 was constructed by inserting synthesized full length SOCS7 cDNA (NM_014598.4) (Jinsirui Biotechnology, Nanjing, China) into BamH1/Xhol sites of pCDNA3.1(+) (Invitrogen).

Huh7.5 cells were seeded in 6-well plates or 96-well plates for 24 h before treatment. Transfection was performed using Lipofectamine^TM^ 2000 (Invitrogen, Carlsbad, CA, USA) according to the manufacturer's instructions. In brief, Oligos, plasmids and Lipofectamine were diluted with serum-free DMEM, respectively. After 5 min at room temperature, each sample was mixed together with Lipofectamine and incubated for 20 min, upon which the mixed solution was applied to the cells dropwise. At 4 h post-transfection, the cells were carefully washed with phosphate buffered saline (PBS), and supplemented with fresh medium. The siRNA and si-NC were diluted to a concentration of 50 nmol/L, and the subsequent treatment was performed at 24 h post-transfection. The transfection efficiency was detected by RT-qPCR.

### Generation of cell culture-derived HCV (HCVcc) and infection assay

Infectious HCV JFH-1 virus was generated from plasmid pJFH-1 (a gift of Dr. Wakita T.) by transfection of an *in vitro* transcribed full-length JFH-1 HCV RNA into Huh7.5 cells, and viral stock solution was prepared by infection of the cells with JFH1 virus, as described by Wakita T. *et al* 29. The HCVcc titration was performed by fluorescence quantitative RT- PCR.

### Quantitative real-time PCR analysis (RT-qPCR)

Total RNA was isolated from cells using TRIzol (Takara, Dalian, China) at 48 h post-treatment. The cDNA was prepared using HiScript 1st Strand cDNA Synthesis Kit (Vazyme, Nanjing, China). The miRNA reverse transcription was separately performed with MicroRNA Reverse Transcription kit (Vazyme, Nanjing, China).

RT-qPCR was performed with SYBR® Premix Ex Taq™ (Takara, Dalian, China) according to the manufacturer's protocol. All primer sequences were shown in Supplementary data 1. Relative abundances of mRNA (or miR-145-5p) to endogenous control GAPDH (or U6) were calculated using the standard “^ΔΔ^Ct method”.

### Dual-luciferase reporter assay

Based on the predictions of bioinformatics, we constructed the sensor vector by joining putative miR-145-5p target binding sequence in UCA1 or SOCS7 with a luciferase reporter vector pmirGLO (Promega) to verify whether miR-145-5p directly binds with UCA1 and SOCS7, respectively.

Huh7.5 cells were seeded in 96-well plates for co-transfection with pmirGLO-UCA1(WT)/pmirGLO-SOCS7(WT) or pmirGLO-UCA1(MUT)/pmirGLO-SOCS7(MUT) reporter plasmid and miR-145-5p mimics (or miR-NC). After 48 h of co-transfection, the luciferase activity was evaluated using the Dual-Luciferase Reporter Gene Assay Kit (Yeasen, Shanghai, China). The firefly luciferase activity was normalized to Renilla luciferase activity.

### Western blot analysis

Huh7.5 cells were lysed using RIPR buffer (Epizyme, Shanghai, China) at 48 h after treatment, and the protein concentration was measured using a BCA protein assay kit. Then the proteins were separated by 12% sodium dodecyl sulfate polyacrylamide electrophoresis (SDS-PAGE) and then transferred onto polyvinylidene fluoride (PVDF) membranes (Millipore, USA). After blocked with 5% non-fat milk for 1 h at room temperature, the membranes were incubated overnight at 4 °C with anti-HCV NS3 antibody (1:1000,Abcam, Cambridge, MA, USA; 1:1000, BioFront, Tallahassee, FL,USA) and anti-GAPDH antibody (1:10000, Abcam, Cambridge, MA, USA). After washed with TBST three times, the membranes were incubated for another 1 h with a secondary goat-anti-mouse IgG antibody conjugated with horseradish peroxidase (HRP) (1:10000, Abcam, Cambridge, MA, USA).Finally, protein bands were detected using the ECL detection reagent (Epizyme, Shanghai, China).

### Nuclear-cytoplasmic fractionation assay

To identify the subcellular localization of UCA1, we performed a nuclear-cytoplasmic fractionation assay using PARIS™ Kit (Invitrogen). When the number of the HCV-infected or mock-infected cells reaches 5×10^6^, the cells were digested with trypsin and harvested by centrifugation at 1,100 rpm for 3 min at 4 °C. After adding ice-cold cell fractionation buffer, the resultant supernatant was carefully aspirated for cytoplasmic RNA assay, and the remaining precipitate was lysed to obtain nuclear RNA *via* ice-cold cell disruption buffer. Eventually, the RNAs of cytoplasmic and nuclear were eluted with elution solution. The expression levels of UCA1 in nucleus and cytoplasm were examined by RT-qPCR.

### Fluorescence *in situ* hybridization (FISH)

FISH was performed using the FISH kit from Servicebio (Wuhan, China).The cells were seeded on slides and subsequently fixed in 4% paraformaldehyde for 20 min after the cell density reached 60-80%. Then the slides were treated with protease K to expose the fragment of nucleic acid and incubated with pre-hybridization solution for 30 min at 60 °C. After discarding pre-hybridization solution, the slides were incubated with hybridization solution with Cy3-labelled UCA1 probe overnight at 30 °C. Subsequently, the slides were incubated with DAPI staining solution for 8 min in the dark followed by discarding hybridization solution, and the results were recorded by fluorescence microscope (Nikon).

### Enzyme linked immunosorbent assay (ELISA)

After 48 h of treatment, the cell culture supernatant was collected and the concentration of IFNs was determined using ELISA. The standards and samples were added to the microtiter plates pre-coated with the specific polyclonal antibody in accordance with the indicated concentration. Then the HRP was added and the plates were incubated for 1 h at 37 °C. Hereafter, the plates were washed 5 times by washing buffer, and the chromogen solution was then added. After incubation in the dark for 15 min, the reaction was stopped by the stop solution and the absorbance of each well was measured at 450 nm wavelength.

### Statistical Analysis

All experiments were independently repeated at least three times, and the graphs were expressed as mean and standard error of mean (Mean ± SEM). Statistical differences between groups were assessed using two-tailed Student's *t*-test by GraphPad8.0 (GraphPad Software, La Jolla, CA, USA). *p*-values lower than 0.05 were regarded as significant.

## Results

### UCA1 was upregulated during HCV infection over time

**Figure 1:**
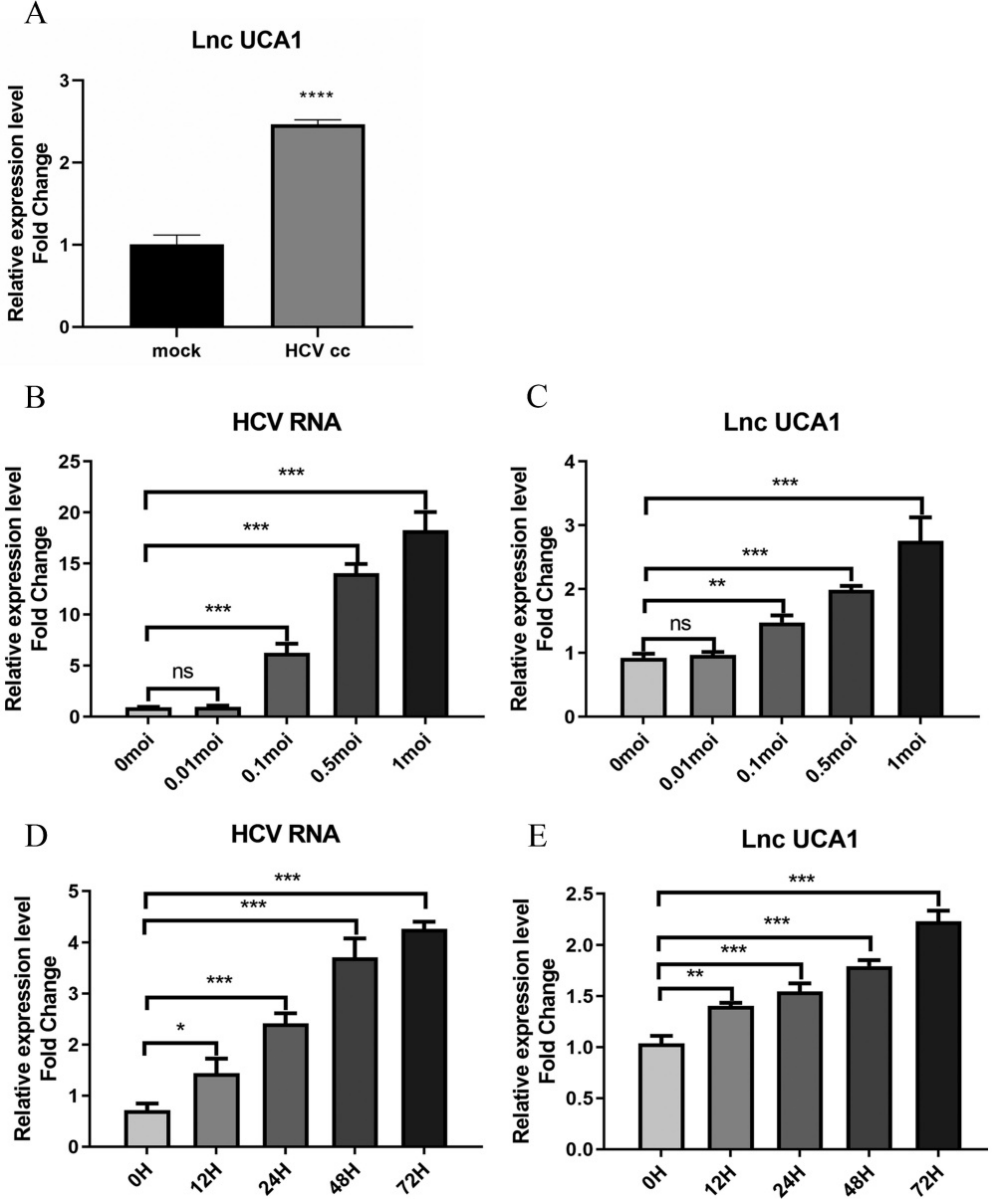
UCA1 was upregulated during HCV infection over concentration and time. Huh7.5 cells were infected with HCV, and then intracellular RNA was harvested to analyze the levels of UCA1 and HCV. (**A**) The expression levels of UCA1 were examined by qRT-PCR in mock-infected and HCV-infected cells. (**B, C**)The expression levels of HCV RNA and UCA1 after HCV infection at different MOIs for 48 h. (**D, E**) The expression levels of HCV RNA and UCA1 after HCV infection at different time points. * *P* < 0.05, ** *P* < 0.01, *** *P* < 0.001, **** *P* < 0.0001, ns., not significant.

To identity the effect of HCV infection on the expression of UCA1, the Huh7.5 cells were infected with the JFH-1 strain (MOI = 0.3) for 6 days. RNAs isolated from HCV-infected and mock-infected Huh7.5 cells were used to evaluate the level of UCA1 by qRT-PCR. As shown in Figure 1A, the expression of UCA1 was significantly upregulated in HCVcc-infected Huh7.5 cells when compared with the mock-infected cells.

To further clarify the correlation between UCA1 and HCV infection, we tested the expression level of UCA1 in Huh7.5 cells infected with HCV at different MOIs as well as different time. The results showed that UCA1 elevated with increasing amounts of MOIs (Fig. 1B and C) and the eminent upregulation was observed from 24 h after infection and onward (Fig. 1D and E). These data suggested that UCA1 expression was stimulated by HCV infection and upregulated during HCV infection over time.

### UCA1 was mainly localized in the cytoplasm

**Figure 2:**
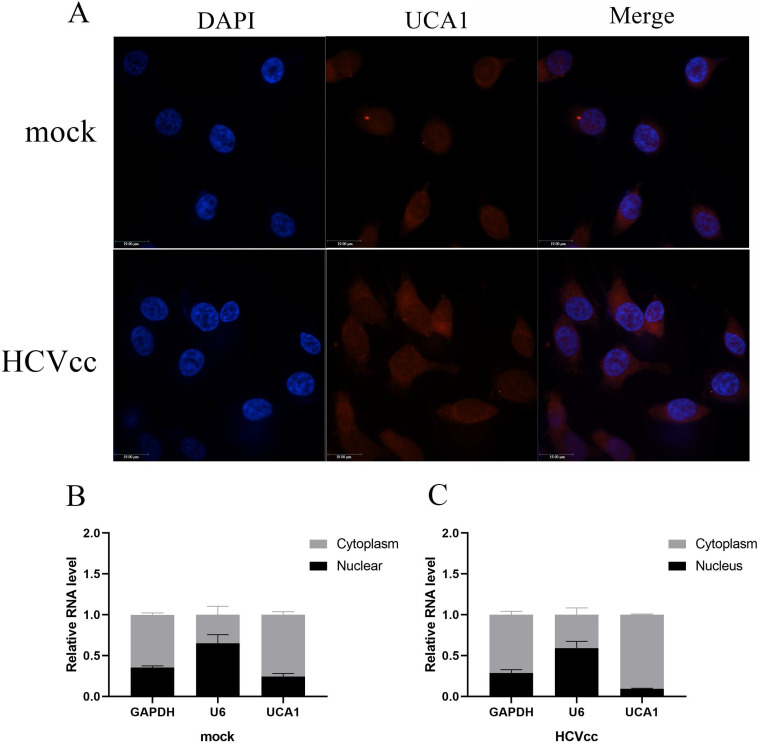
UCA1 was predominately located in the cytoplasm. (**A**) FISH analysis in Huh7.5 cells processed as previously described in methods. Cy3 labeled probe stained red, DAPI-stained nuclei blue. Scale bar: 19 µm. (**B, C**) The expression levels of U6, GAPDH and UCA1 were assessed by RT-qPCR in the nuclear and cytoplasmic fractions 48 h post-infection. U6 was detected as the nuclear transcript control, and GAPDH as the cytoplasmic transcript control.

To gain insight into the underlying mechanism of UCA1 in regulating HCV replication, we subsequently examined its cellular localization using FISH. The uninfected and infected Huh7.5 cells were fixed and hybridized with a full length anti-sense UCA1 probe labeled with cy3 for further observation. As can be seen in Fig. 2A, UCA1 was upregulated and predominantly localized in the cytoplasm after HCV infection compared with mock-infected group.

To confirm this result, we performed nuclear-cytoplasmic separation assay. As expected, the results of RT-qPCR showed that GAPDH transcripts accumulated mainly in the cytoplasm in both treated and untreated group. On the contrary, the expression level of U6 in the nucleus was significantly higher than that of the cytoplasm. Importantly, UCA1 dominantly localized in the cytoplasm, and highly increased in the infected cells compared with the uninfected cells (Fig. 2B and C).

### UCA1 knockdown inhibited HCV infection and upregulated the expression of IFNs and ISGs

**Figure 3:**
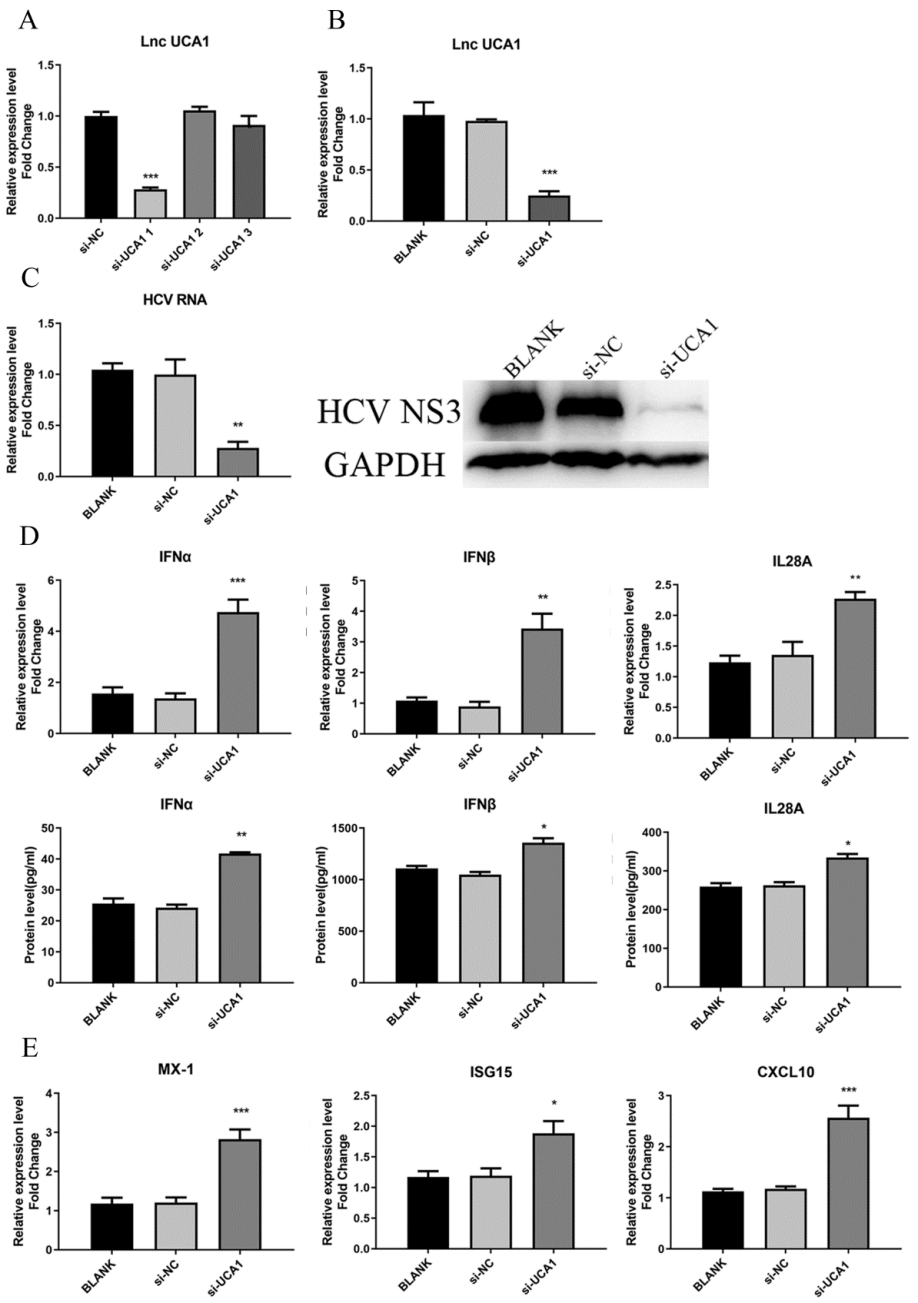
UCA1 knockdown inhibited HCV infection and upregulated the expression of IFN and ISGs. (**A, B**) The relative expression levels of UCA1 in Huh7.5 cells transfected with si-UCA1#1, si-UCA1#2 or si-UCA1#3 were examined by qRT-PCR (si-UCA1#1 is referred to as si-UCA1 afterwards). (**C**) The changes of HCV RNA and NS3 protein were respectively evaluated by qRT-PCR and western bolt after UCA1 expression was knocked down. (**D, E**) The mRNA expression levels of IFN and ISGs were tested at 48 h post-transfection. * *P* < 0.05, ** *P* < 0.01, *** *P* < 0.001 *vs* si-NC.

As for the loss-of-function assay, small interfering RNAs (siRNAs) specifically targeting UCA1 transcripts were used to test their efficiency to downregulate UCA1. Huh7.5 cells were transfected with siRNAs (si-UCA1#1, si-UCA1#2, si-UCA1#3 or negative control si-NC). We analyzed their knockdown efficiency by detecting the expression level of UCA1 at 48 h post-transfection. The results showed that si-UCA1#1 had higher interference efficiency than si-UCA1#2 and #3, so si-UCA1#1 was chosen for the subsequent experiments (Fig. 3A and B).

To investigate the impact of UCA1 on HCV infection, Huh7.5 cells were transfected with si-UCA1#1 or si-NC and subsequently infected with HCVcc (MOI=0.3). Total RNA and HCV NS3 protein were extracted to assess the impact of UCA1 knockdown on HCV infection. The results told that the titer of HCV was robustly decreased in the group of UCA1 knockdown (Fig. 3C), indicating that HCV replication was suppressed when the cellular UCA1 was knocked down.

To determine whether the inhibitory effect of UCA1 knockdown on HCV replication was dependent on elevated antiviral response, we tested the levels of some virus-related IFNs and interferon-stimulated genes (ISGs).The changes were shown in Fig. 3D and E, which displayed elevated expression levels of IFNs and ISGs. These results indicated that knockdown of UCA1 significantly inhibited HCV replication probably by increasing antiviral response in Huh7.5 cells.

### UCA1 targeted and negatively regulated miR-145-5p level

**Figure 4:**
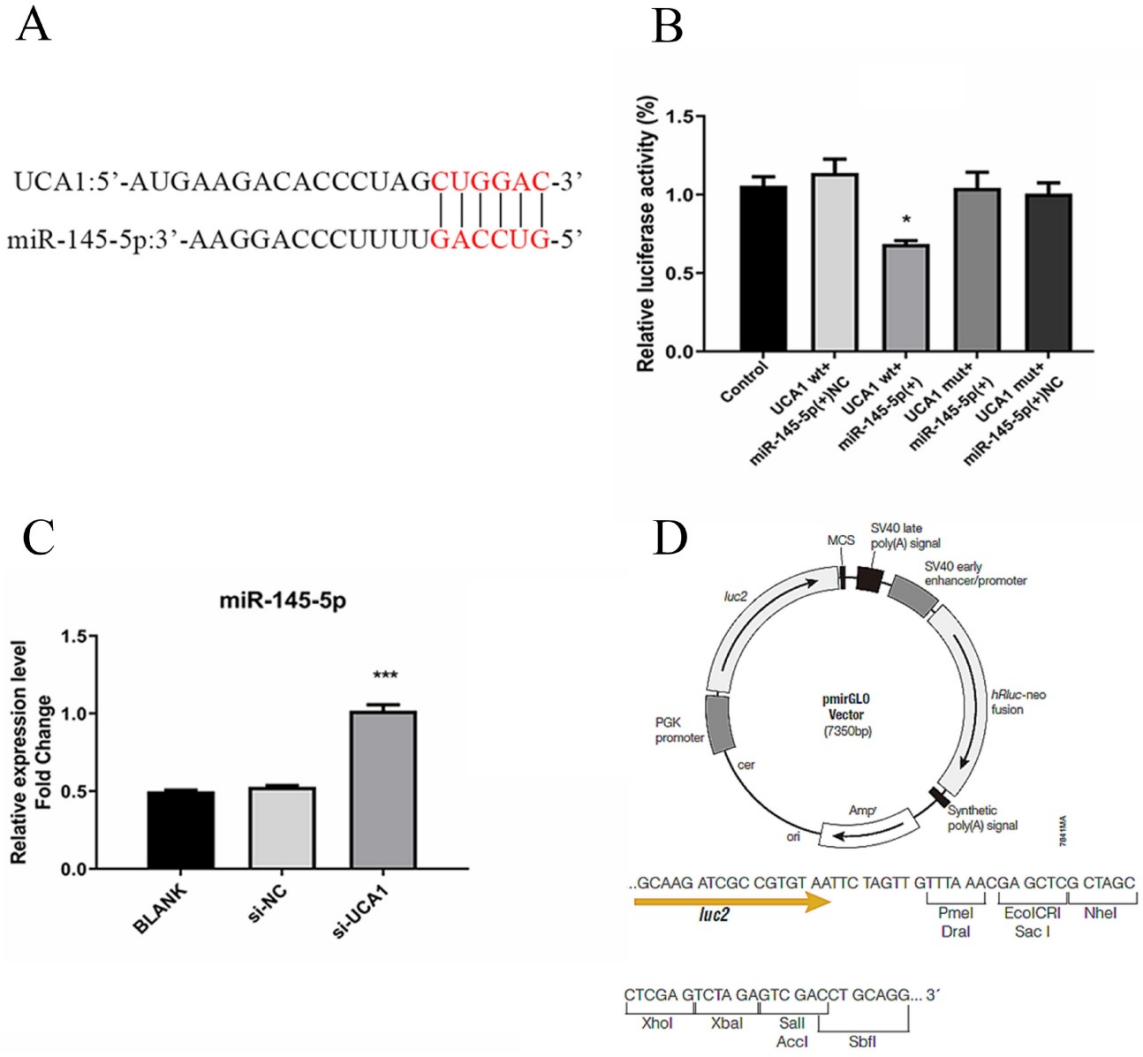
UCA1 targeted and negatively regulated miR-145-5p. (**A**) The 3′-UTR of UCA1 contained the predicted binding site for miR-145 according to the results of bioinformatics analysis. (**B**) Reporter assays displayed that miR-145-5p significantly decreased the luciferase activity of the pmirGLO-UCA1 (WT) instead of the mutant. (**C**) The expression of miR-145-5p was assayed after the UCA1 was knocked down via si-UCA1. (**D**) Schematic diagram of backbone vector pmirGLO (Promega). * *P* < 0.05 *vs* Control; *** *P* < 0.001 *vs* si-NC.

LncRNAs regulate cancer progression partly by participating in the competitive endogenous RNA regulatory network. Based on bioinformatics analysis, the 3′-UTR of UCA1 included a putative binding site for miR-145-5p (Fig. 4A). The expression level of miR-145-5p was upregulated with UCA1 knockdown in the infected Huh7.5 cells (Fig. 4B). These results showed that UCA1 might play a role in the deregulation of miR-145-5p. For further confirmation of the interaction, pmirGLO-UCA1(WT) and pmirGLO-UCA1(MUT) were conducted. pmirGLO-UCA1(WT) or pmirGLO-UCA1(MUT) was co-transfected with miR-145-5p mimics or miR-NC into cells, respectively.

Our data showed that the co-transfection of miR-145-5p mimic and pmirGLO-UCA1 (WT) significantly reduced the luciferase activities of the target sequence of UCA1, whereas those of the mutant sequence of UCA1 showed no detectable changes (Fig. 4C). These results suggested that UCA1 can directly target miR-145-5p in Huh7.5 cells, which may act as a sponge of miR-145-5p.

### miR-145-5p showed inhibitory effect on HCV infection

**Figure 5:**
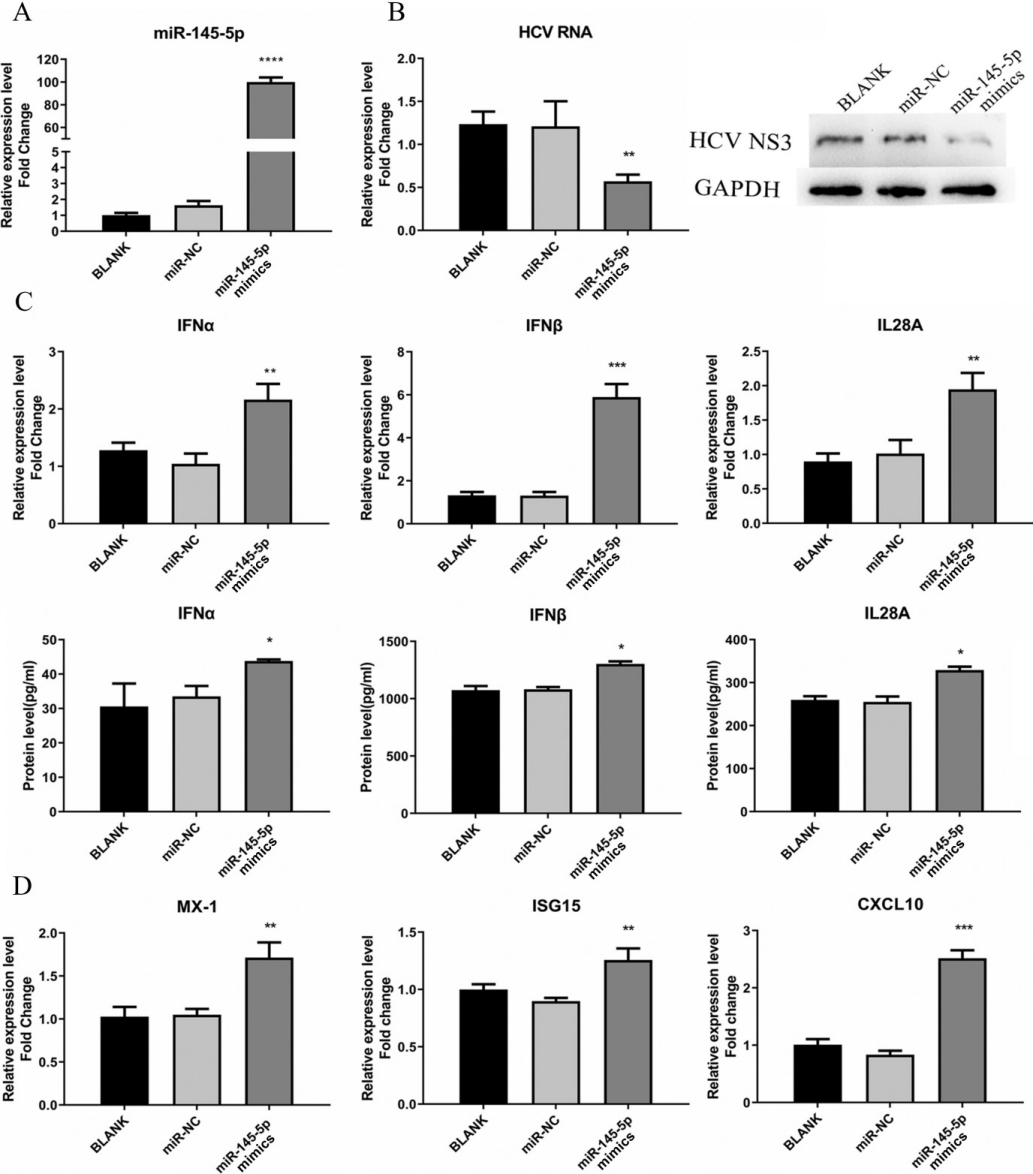
miR-145-5p showed inhibitory effects on HCV infection. (**A**) The miR-145-5p was overexpressed by transfection of miR-145-5p mimics. (**B**) The expression levels of HCV RNA and NS3 protein were analyzed by qRT-PCR and western blot after transfection of miR-145-5p mimics. (**C, D**) The expression levels of IFNs, MX-1, ISG15 and CXCL10 was evaluated by qRT-PCR and ELISA under the condition that miR-145-5p was overexpressed. * *P* < 0.05, ** *P* < 0.01, *** *P* < 0.001, **** *P* < 0.0001 *vs* miR-NC.

To determine whether the expression of miR-145-5p affects HCV infection, we adopted synthesized miR-145-5p mimics to overexpress miR-145-5p in cells. Huh7.5 cells were transfected with miR-145-5p mimics or miR-NC and subsequently infected with HCVcc. As shown in Fig. 5A, overexpression of miR-145-5p made Huh7.5 cells less susceptible to HCV infection, displaying a significant reduction of both HCV RNA and viral NS3 protein expression levels (Fig. 5B).

Meanwhile, we tested the responses of IFN and ISGs in the cells transfected with miR-145-5p mimics or miR-NC. We found that the expression levels of IFN and ISGs were significantly increased when cellular miR-145-5p was over-expressed, compared with the control group (Fig. 5C and D). The data displayed that miR-145-5p positively regulated antiviral response in HCV-infected Huh7.5 cells.

To further confirm the inhibitory effect of miR-145-5p on HCV infection, we decreased the expression of miR-145-5p in Huh7.5 cells by transfecting miR-145-5p inhibitor. We found that the concentrations of HCV RNA and HCV NS3 protein were higher in the group of cells transfected with miR-145-5p inhibitor when compared with NC group (Fig. S1A). These results had shown that the lower level of miR-145-5p was more beneficial for HCV infection (Fig. S1B,C and D).

### miR-145-5p could target SOCS7 to regulate antiviral responses

**Figure 6:**
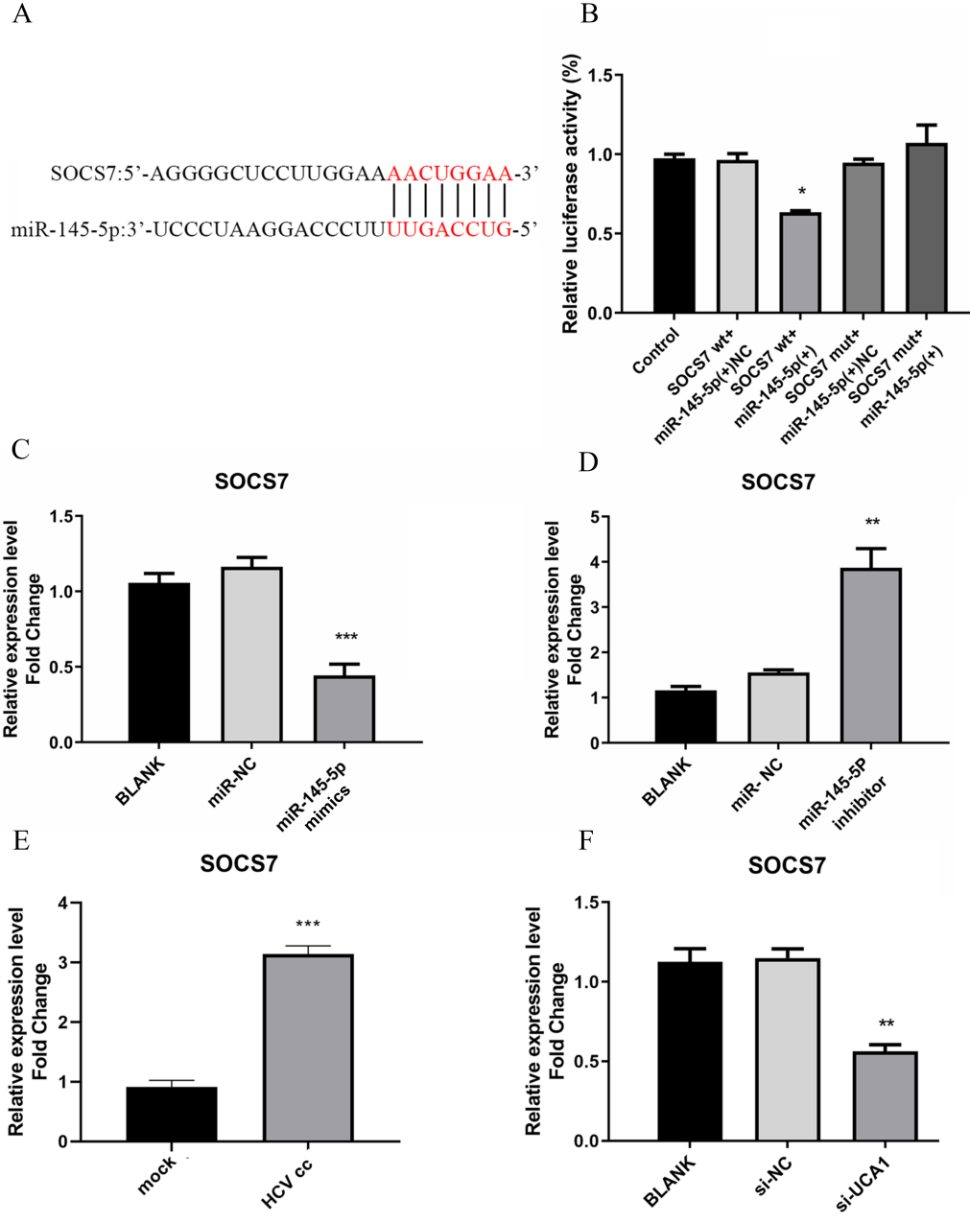
miR-145-5p can target SOCS7 to regulate antiviral response. (**A**) The complementary regions of the 3`-UTR of SOCS7 mRNA to miR-145-5p. (**B**) The luciferase activities were detected after co-transfection of miR-NC or miR-145-5P mimics with pmirGLO-SOCS7(WT) or pmirGLO-SOCS7 (MUT). (**C, D**) The relative expression level of SOCS7 RNA was analyzed after the expression of miR-145-5p was increased or decreased. (**E**) HCV infection upregulated the expression of SOCS7 RNA when compared with mock-infection. (**F**) The RNA level of SOCS7 was determined in Huh7.5 cells transfected with si-UCA1 or si-NC. * P < 0.05 vs Control; ** *P* < 0.01, *** *P* < 0.001 vs miR-NC; *** *P* < 0.001vs mock; ** *P* < 0.01 vs si-NC.

To identity the target genes of miR-145-5p in Huh7.5 cells, we used miRTarBase to predict some candidate genes associated with antiviral response. Then we focused on suppressor of cytokine signaling 7 (SOCS7) as a possible target gene with a potential binding site of miR-145-5p, as shown in Fig. 6A. As a member of SOCS protein family, it has been reported to be associated with induction of IFN and ISGs 24, 25.

To further verify whether miR-145-5p can bind directly with SOCS7, the assay for dual-luciferase activity was performed. pmirGLO-SOCS7(WT) or pmirGLO-SOCS7(MUT) and miR-145-5p mimics or NC were co-transfected into Huh7.5 cells, and then luciferase activities were tested. The results demonstrated that the luciferase activities of Huh7.5 cells co-transfected with pmirGLO- SOCS7(WT) and miR-145-5p mimics was notably reduced compared to the other groups (Fig. 6B), which implied that miR-145-5p could bind directly with SOCS7 to modulate antiviral response in Huh7.5 cells.

To detect the effect of miR-145-5p on the expression of SOCS7, Huh7.5 cells were transfected with miR-145-5p mimics or inhibitor, respectively. These results showed that SOCS7 was robustly suppressed in the condition of miR-145-5p overexpression (Fig. 6C and D), indicating miR-145-5p could specifically target SCOS7 to decrease its expression level. Then we further examined the level of SOCS7 in Huh7.5 cells infected with HCVcc and transfected with si-UCA1, respectively. Our data had shown that the expression of SOCS7 was increased followed by HCV infection. Conversely, in the case of UCA1 knockdown, SOCS7 expression was suppressed (Fig. 6E and F), indicating that UCA1 regulated SOCS7 expression via miR-145-5p.

### SOCS7 was favorable to HCV replication by suppressing response of IFNs

**Figure 7:**
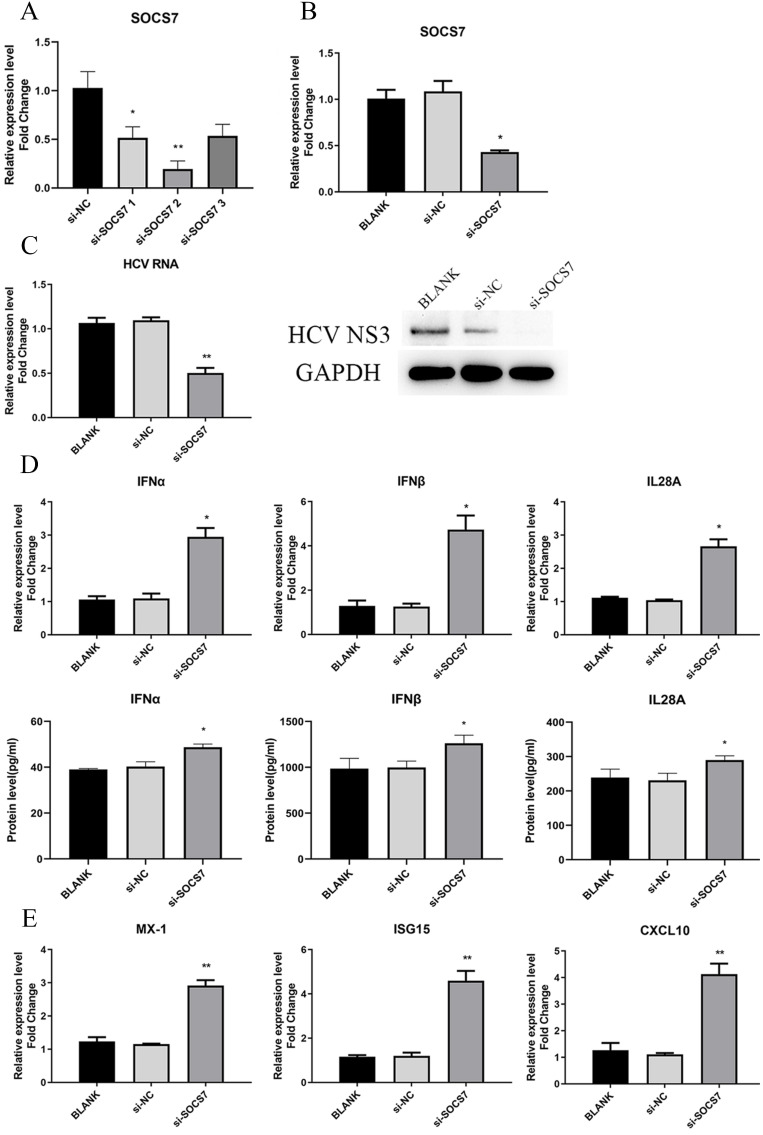
SOCS7 favored to HCV replication by suppressing response of IFNs. (**A, B**) The RNA level of SOCS7 was assessed by qRT-PCR in Huh7.5 cells transfected with si-SOCS7#1, si-SOCS7#2 or si-SOCS7#3. (si-SOCS7 refers to si-SOCS7#2 afterwards). (**C**) The levels of HCV RNA and NS3 protein were determined after SOCS7 was downregulated. (**D, E**) At 48 h post-transfection of si-SOCS7, the expressions of IFNs and several ISGs were evaluated. * *P* < 0.05, ** *P* < 0.01, *vs* si-NC.

To determine the function of SOCS7 in HCV replication and antiviral response, we downregulated the level of SOCS7 through transfection of si-SOCS7 into Huh7.5 cells, which were subsequently infected with HCVcc. At 48 h post-infection, the intracellular RNA and proteins were harvested to examine the expression of SOCS7, IFNs, and HCV, etc. Our data displayed that HCV replication was significantly inhibited (Fig. 7C), and the antiviral response was obviously enhanced (Fig. 6D and E) on condition that SOCS7 expression was decreased.

To further confirm the results, we overexpressed SOCS7 by transfection of pcDNA3.1-SOCS7 construct. The conclusion drawn was consistent with the previous results, indicating that SOCS7 had an inhibitory effect on antiviral response and hence promoted the replication of HCV (Fig. S2).

### UCA1 modulated HCV infection and antiviral response by repressing the expression of miR-145-5p

**Figure 8:**
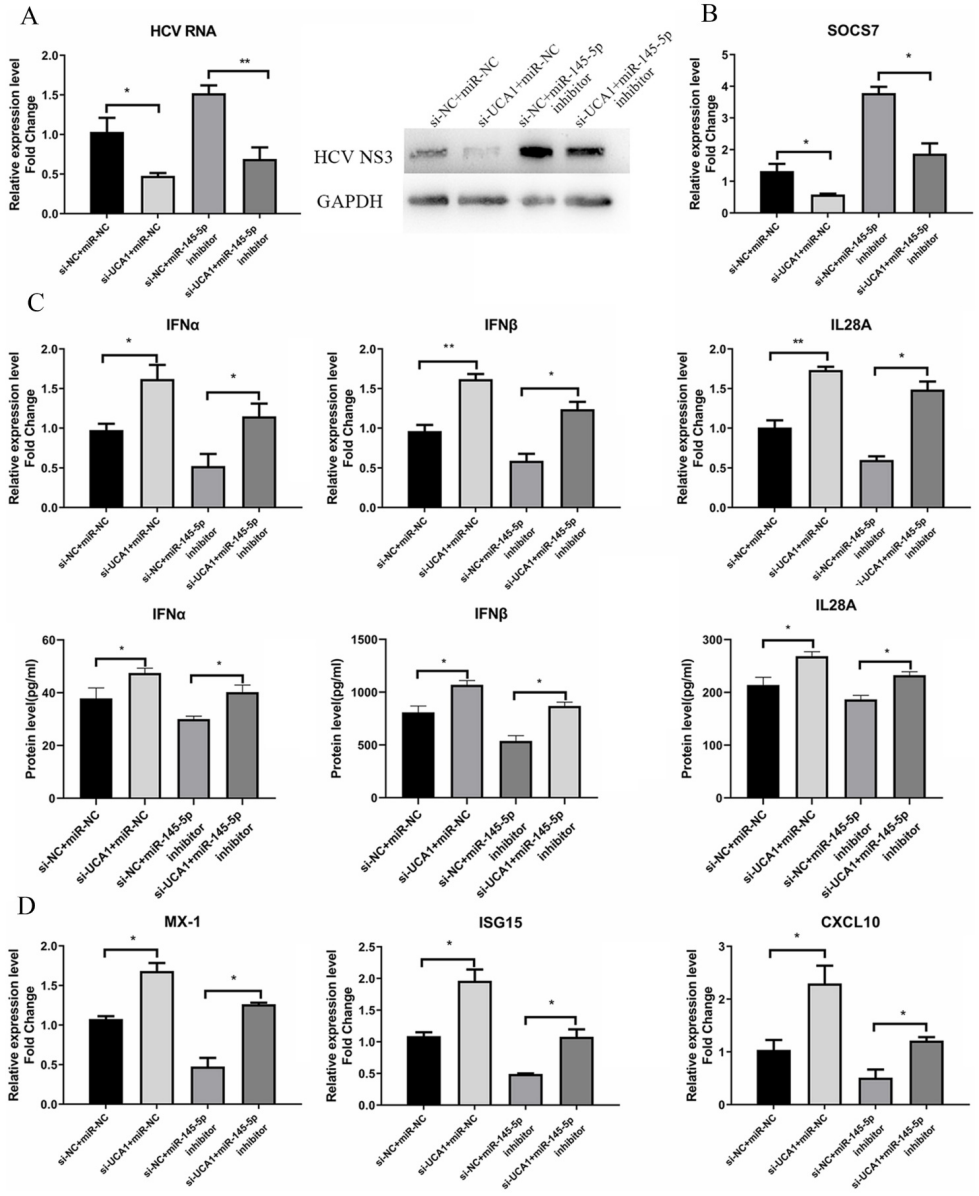
UCA1 modulated HCV infection and antiviral response by repressing the expression of miR-145-5p. (**A**) Huh7.5 cells were transfected with si-UCA1 or si-NC, and then transfected with miR-NC or miR-145-5p inhibitor 24 h later. The HCV replication was evaluated by qRT-PCR and western bolt. (**B**) The SOCS7 expression was quantitated by qRT-PCR in Huh7.5 cells transfected with miR-NC or miR-145-5p inhibitor and si-NC or si-UCA1. (**C,D**) Responses of IFNs and ISGs were assessed at 48 h post-treatment. * *P* < 0.05, ** *P* < 0.01.

To further investigate whether miR-145-5p is involved in the promoting effect of UCA1 on HCV infection, we conducted transfection combinations prior to the assessment of HCV infection. Western blot assay revealed that the viral NS3 protein expression was significantly down-regulated in the UCA1 (-) + miR-145-5p(+) group and up-regulated in the UCA1 (+) + miR-145-5p (-) group. In the UCA1(-) + miR-145-5p(-) group, the miR-145-5p inhibition rescued the inhibitory effect of UCA1 knockdown on HCV NS3 protein expression. The RT-qPCR results of HCV RNA level were similar to the above results (Fig. 8A). Taken together, our data indicated that UCA1 modulated HCV infection by repressing the expression of miR-145-5p.

Meanwhile, the antiviral response was also tested. The data showed that the expression levels of IFNs and ISGs were highest in cells co-transfected with si-UCA1 and miR-NC than those in the other groups, while SCOS7 expression was robustly inhibited in the UCA1 (-) + miR-145-5p (+) group (Fig. 8B), which suggested that UCA1 knockdown or miR-145-5p overexpression can enhance antiviral response in Huh7.5 cells infected with HCV by down-regulating expression of SOCS7 (Fig. 8C and D).

## Discussion

Accumulating researches have shown that UCA1 is dysregulated in a variety of cancer tissues and cells. It can target a variety of signaling pathways to regulate the occurrence and development of diseases 13, 30, but there are few studies on its regulatory effect on virus replication. In the study of Sunantha Sethuraman *et al.*, they discovered that UCA1 was upregulated by the Kaposi's sarcoma-associated herpesvirus (KSHV) latency-associated proteins Kaposin and vCyclin in the cells infected with KSHV 13, 31. Moreover, the elevated UCA1 facilitated the proliferation and migration of Kaposi's sarcoma (KS) cells, which likely contributed to KSHV pathogenesis and tumorigenesis 31. Therefore, this indicates that UCA1 also plays an important role in virus replication.

In the present study, we demonstrated that the expression of UCA1 was upregulated in Huh7.5 cells infected with HCV over concentration and time. Moreover, knockdown of UCA1 was shown to suppress HCV infection and elevate antiviral response. Our FISH detection showed that UCA1 was mainly localized in the cytoplasm, which may function as a ceRNA to sponge a specific miRNA to regulate HCV infection and IFN response. The results of qRT-PCR and luciferase reporter assay defined that UCA1 could bind directly with miR-145-5p to mediate HCV replication and antiviral response.

Interestingly, Kosuke Horita *et al*. found that the expression of UCA1 was dysregulated in human colorectal cancer cells infected with oncolytic vaccinia virus (OVV), and it targeted miR-18a or miR-182, resulting in increased expression and activation of Cdc42^32^. Cdc42, as a kind of GTPases, belongs to Rho family, which has important regulatory effects on cell adsorption, migration, the formation and disassembly of cell actin cytoskeleton 33. In OVV-infected cells, activated Cdc42 promoted the formation of filopodia and ultimately facilitated virus cell-to-cell spread, but had no significant effect on the adsorption, entry and replication of virus 32. Moreover, Cdc42 has been confirmed to be positively correlated with the infectivity of HCV 34. Therefore, in HCV-infected Huh7.5 cells, UCA1 may not only regulate the antiviral response, but also regulate the expression of Cdc42 to enhance the infectivity of HCV, but it needs to be further verified.

We selected the downstream target molecules of UCA1 through bioinformatics, and then verified it with dual-luciferase assay system. Our data displayed that UCA1 directly targeted miR-145-5p and inhibited the biological function of miR-145-5p. In addition, we found that miR-145-5p could inhibit the replication of HCV by promoting intracellular antiviral responses.

Additionally, we found that miR-145-5p could target 3' UTR region of SOCS7 mRNA and downregulate its expression level to increase the induction of IFN induction. Meanwhile, our data shown that in the case of HCV infection, both UCA1 and SOCS7 will be up-regulated. On the contrary, the knockdown of UCA1 would reduce the mRNA level of SOCS7, indicating that HCV infection and the up-regulation of UCA1 would simultaneously promote SOCS7 transcription, ultimately leading to an increase in SOCS7 mRNA levels. We further verified that SOCS7 exerted an important regulatory effect on IFN response and ISGs production through the SOCS7 knockdown experiment, which was probably achieved via the signal of the Janus kinase-signal transducer and activator of transcription (Jak-STAT) pathway. The binding of IFN to its receptor (IFNR) activates the Jak-STAT pathway 35, 36, but the upregulated SOCS7 inhibits IFN response, thereby indirectly inhibiting the phosphorylation of Jak and STATs. The phosphorylated STATs will form dimers and enter the nucleus to bind to specific DNA sequences in the promoter region of the target gene, and then initiate the transcription of the target gene, such as ISGs 36. Finally, ISGs encode a wide range of cytokines, including ISG15, myxovirus resistance A(MX-1), and protein kinase R, etc., promoting the anti-virus defenses^37^.

The ubiquitin-like protein ISG15 is an interferon-induced protein and is considered to be a major participant in the host's antiviral response. Accumulative evidence shows that unbound ISG15 can regulate viral replication and host response through non-covalent protein interactions and its role as cytokines 38. The interferon-induced MX-1 protein exists in almost all vertebrates. It is an evolutionarily conserved dynein-like large GTPase, which can significantly inhibit a variety of RNA viruses and also has a certain inhibitory effect on other viruses 39. CXCL10, also known as IFN-γ-inducible protein 10, is significantly dysregulated during HCV infection, and the interaction between CXCL10 and its receptor CXCR3 can regulate the occurrence and development of viral hepatitis 40, 41. This means that CXCL10 may be involved in the antiviral response of patients with hepatitis C. Our results have also shown that CXCL10 exerted an inhibitory effect on HCV replication.

Increasing evidence has shown that cytokine-induced signal transduction pathways can be regulated by SOCS protein family, consisting of SOCS1-SOCS7 and CIS 25. Moreover, the expression of the SOCS proteins can be induced in response to cytokine stimulation, and the overexpression of SOCS proteins has an inhibitory effect on signal transduction via the releases of lots of cytokines in various cell lines, suggesting that SOCS proteins may be important for the modulation of IFN response 24, 25. Until now, the functions of CIS, SOCS1, SOCS2, and SOCS3 proteins are well clarified 25, 42, 43. For instance, Krebs *et al* found that SOCS3 could regulate fetal liver erythropoiesis likely through Epo signaling* in vivo*, and SOCS1 negatively regulated the response of IFN-γ signaling and the differentiation of T cell 25. Similarly, SOCS2 and CIS have been found to inhibit the signaling such as GH, insulin-like growth factor 1 (IGF-1) and prolactin 43-45. Our data has shown that SOCS7 could exert a suppressive effect on IFN response, which may be conducive to viral infection.

Therefore, our results explain that HCV infection leads to the up-regulation of UCA1 expression, which, together with HCV, increases SOCS7 mRNA level. Valerio Pazienza *et al* found that genotype 3a HCV core protein induced the expression of SOCS7 by inhibiting the activation of peroxisome proliferator activated receptor gamma (PPAR-γ) in Huh7.5 cells 46. Consequently, in genotype 2a HCV JFH1 infected Huh7.5 cells, by up-regulated UCA1 binding competitively to miR-145-5p, HCV increases SOCS7 expression through inhibiting miR-145-5p-mediated SOCS7 mRNA silencing. Whether HCV Core protein can increase SOCS7 expression through other mechanisms requires further investigation.

**Figure 9:**
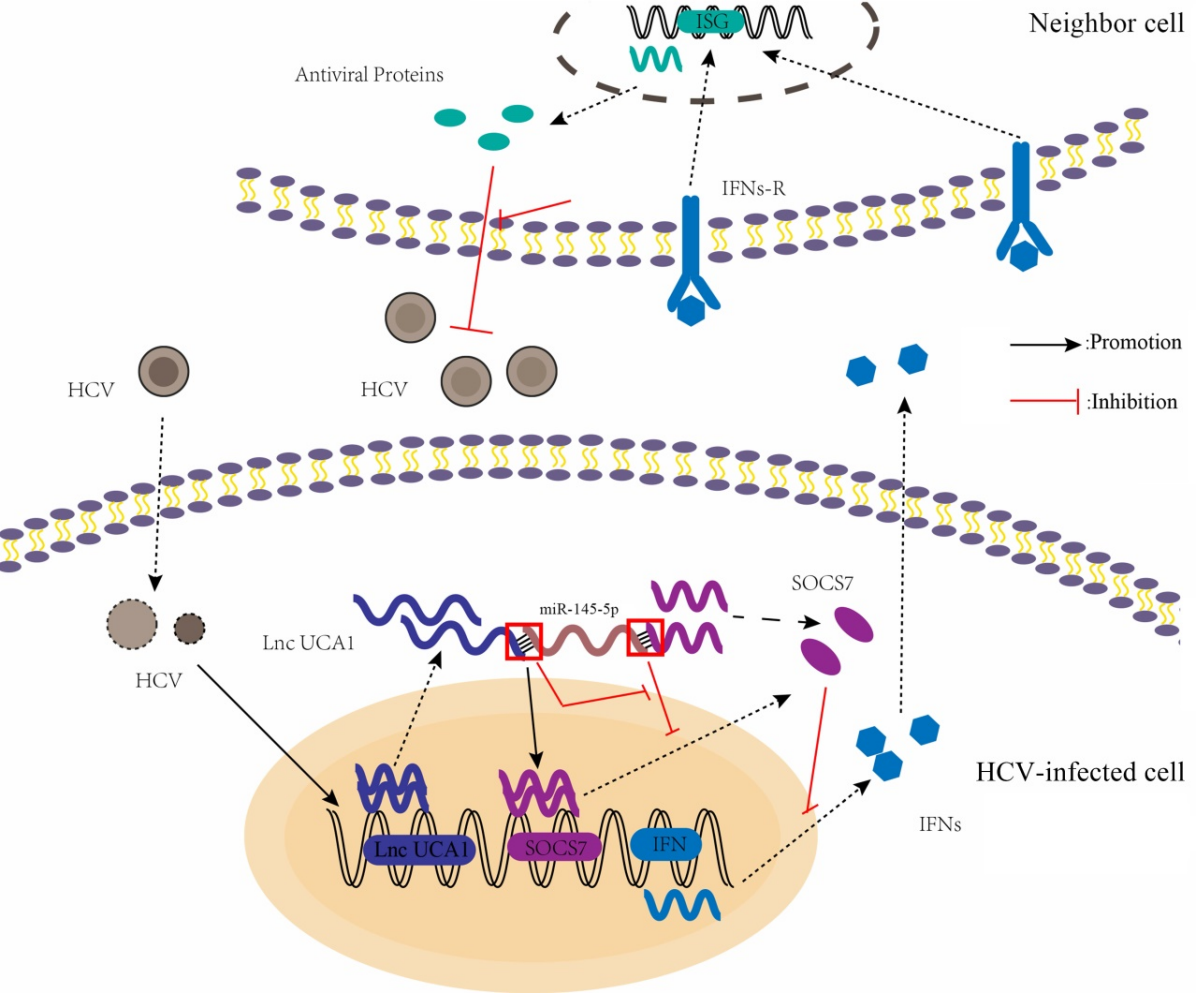
A proposed model illustrating that UCA1 regulates HCV replication and antiviral response via miR-145-5p/SOCS7/ IFN pathway. HCV infection upregulates UCA1 expression, which directly targets and negatively regulates miR-145-5p, leading to the increased level of SOCS7 and consequently decreased secretion of IFN. Decrease of interferon expression results in reduced interferon binding to receptors on the surface of neighbor cells and reduced activation of interferon receptors. Ultimately , the antiviral response is inhibited and HCV replication is promoted.

In conclusion, we present the first evidence that UCA1 promotes HCV replication in Huh7.5 cells through negatively regulating the level of miR-145-5p, and subsequently inhibiting the expression of SOCS7 and its antiviral responses (Fig. 9). The findings from this study could contribute to the comprehensive understanding of the virus-host interaction, though the precise mechanism underlying HCV stimulates UCA1 expression is to be analyzed.

## Supplementary Material

Supplementary figures.
