# Supplementary material for: Long noncoding RNA UCA1 regulates HCV replication and antiviral response via miR-145-5p/SOCS7/IFN pathway
Source: Int J Biol Sci. 2021 Jul 5;17(11):2826–40. doi: 10.7150/ijbs.59227 (PMC8326114; doi:10.7150/ijbs.59227)
Supplement: Supplementary file 1 — Supplementary figures. [file ijbsv17p2826s1.pdf]

## Supplementary Materials

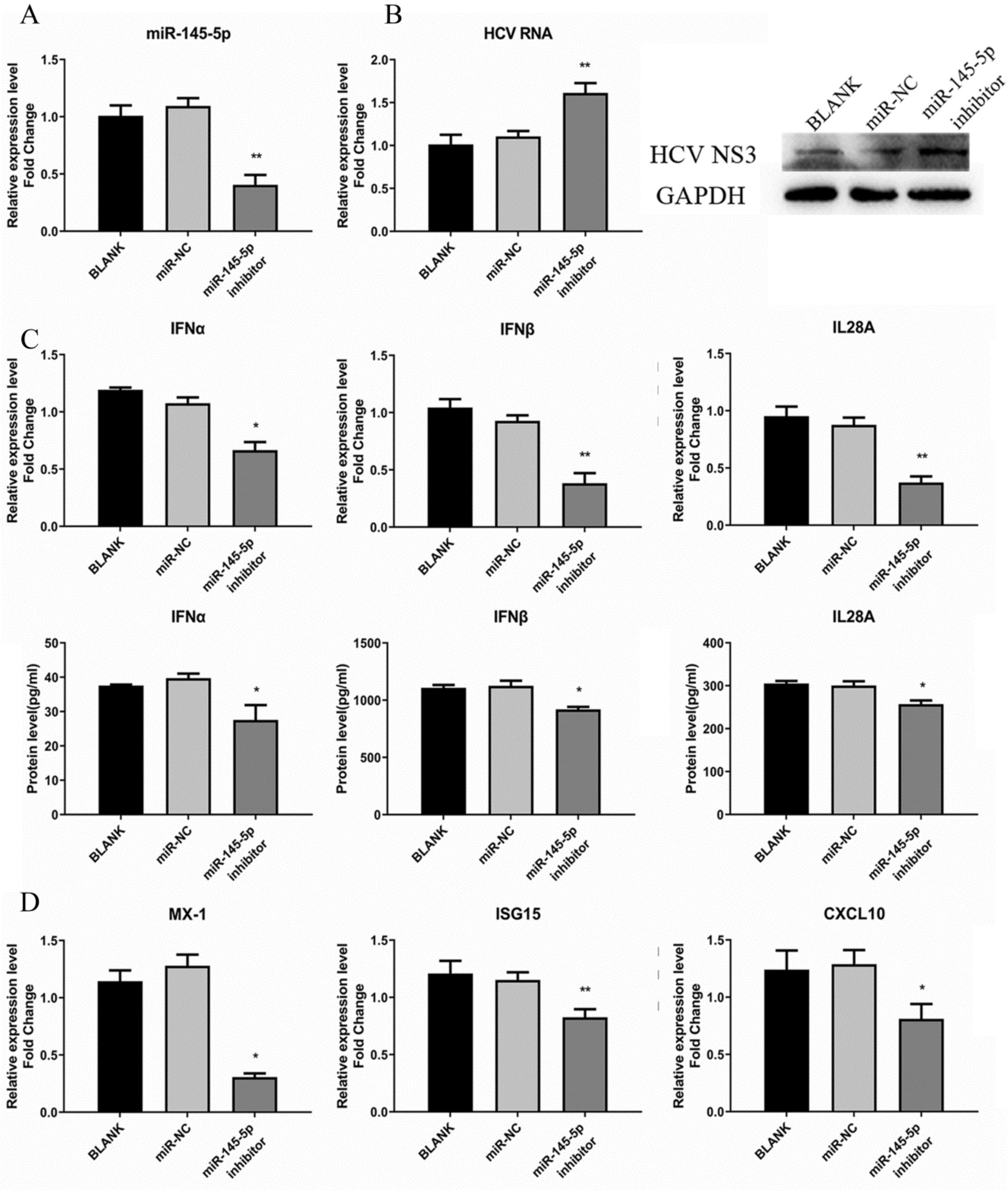


**Figure S1**. Inhibition of miR-145-5p expression suppressed antiviral response. (**A)** The expression of miR-145-5p was detected after 48h of transfection with miR-145-5p inhibitor or miR-NC.(**B)** qRT-PCR and western bolt were performed to measure the levels of HCV RNA and NS3 protein. (**C, D)** Antiviral response was determined by qRT-PCR and ELISA on condition that miR-145-5p expression was inhibited. * $P < 0.05$, ** $P < 0.01$*vs* miR-NC.

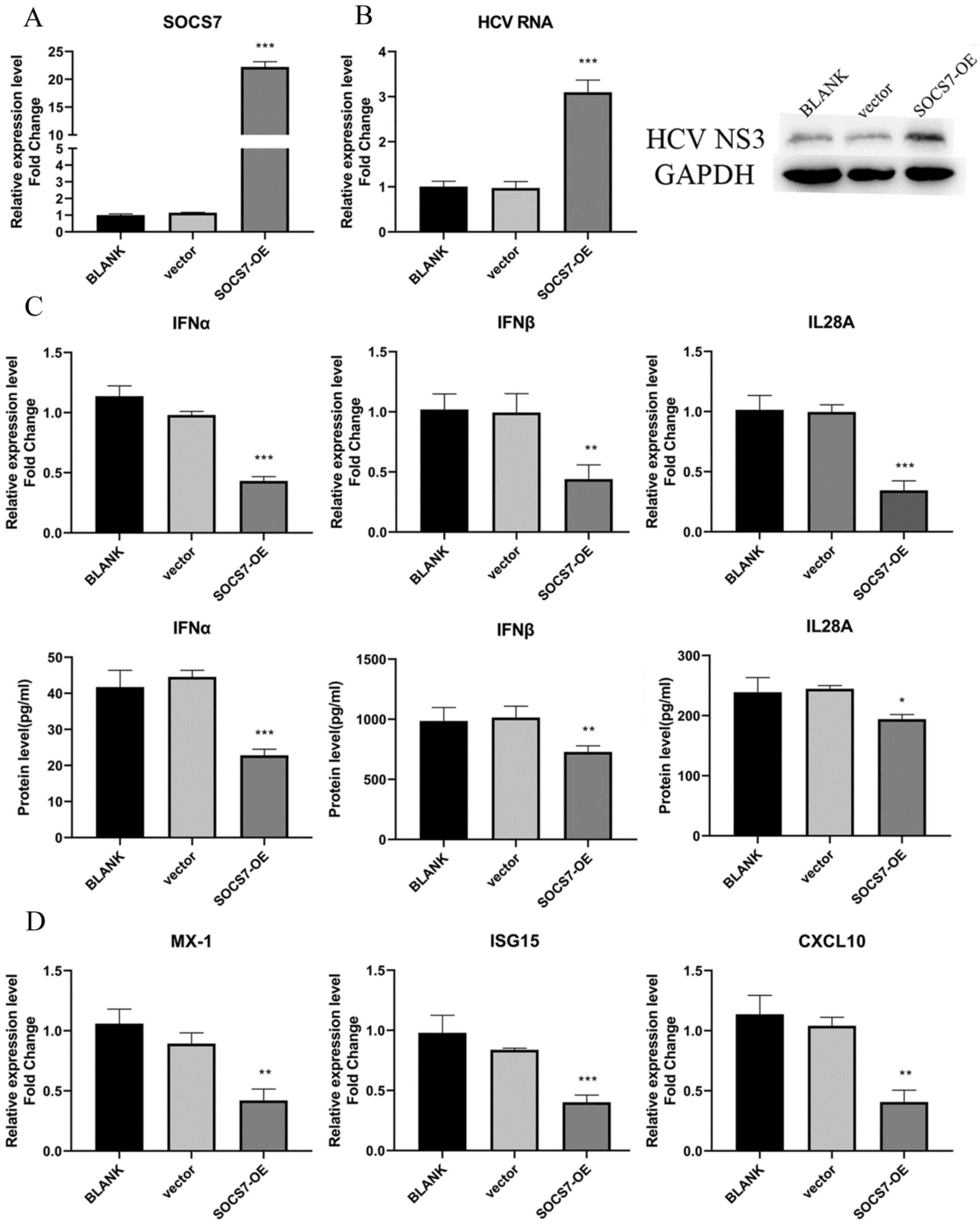


**Figure S2.** SOCS7 overexpression inhibited the antiviral response and finally promoted HCV replication. (**A)** The mRNA level of SOCS7 was quantitated in Huh7.5 cells transfected with SOCS7 overexpression (SOCS7-OE) construct pcDNA3.1-SOCS7. (**B, C** and **D)** HCV replication and antiviral response were analyzed 48 h after SOCS7 overexpression. * $P < 0.05$, ** $P < 0.01$, *** $P < 0.001$ *vs* vector.

**Table S1.** Primers of qRT-PCR

| **Target Gene** | **Primer Sequences (5` -3`)** |
|---|---|
| GAPDH | F:CTGCCAACGTGTCAGTGGTG<br>R: TCAGTGTAGCCCAGGATGCC |
| HCV 5`-UTR | F: CGG GAG AGC CAT AGT GG<br>R: AGT ACC ACA AGG CCT TTC G |
| UCA1 | F: CGGGTAACTCTTACGGTGGA<br>R: ATGGGCATGGCTTTATTCTG |
| miR-145-5p | F: TGTCCTCACGGTCCAGTTTT<br>R: ACCATGACCTCAAGAACAGTATT |
| SOCS7 | F: AAACTGTGTCGCTTGTGGATG<br>R: GAGGTAGGGGGAAAGACTGC |
| IFNα | F: CAGAGTCACCCATCTCAGCAAGC<br>R: CACCACCAGGACCATCAGTAAAGC |
| IFNβ | F: GCACAACAGGTAGTAGGCGACAC<br>R: CAATGCGGCGTCCTCCTTCTG |
| IL28A | F: GCCCTGACGCTGAAGGTTCTG<br>R: GCGGAAGAGGTTGAAGGTGACAG |
| MX-1 | F: TGCATCGACCTCATTGACTC<br>R: ACCTTGCCTCTCCACTTATC |
| ISG15 | F: ACTCATCTTTGCCAGTACAGGAG<br>R: CAGCATCTTCACCGTCAGGTC |
| CXCL10 | F: GTGGCATTCAAGGAGTACCTC<br>R: TGATGGCCTTCGATTCTGGATT |

**Table S2.** UCA1 probe used in FISH

| |
|---|
| UCA1 probe: 5’-CY3-ATGGGCATGGCTTTATTCTGGAATGGTGAA-3’ |

**Table S3.** siRNA sequences of UCA1 and SOCS7

| **siRNA** | **Sequence(5`-3`)** |
|---|---|
| si-UCA1#1 | CCATCAGATCCTTGCCCAT |
| si-UCA1#2 | GGTAATGTATCATCGGCTT |
| si-UCA1#3 | GCTAACTGGCACCTTGTTA |
| si-SOCS7#1 | GAGCCTCAGTTTCCGATCA |
| si-SOCS7#2 | GGACCGCTGTCAATCTGTT |
| si-SOCS7#3 | GAGGTATACCTGTCTCTAA |

**Table S4.** Primers of miR-145-5p mimics and inhibitor

| Primers | Sequences (5`-3`) |
|---|---|
| hsa-miR-145-5p mimics | F:GUCCAGUUUUCCCAGGAAUCCCU<br>R:AGGGAUUCCUGGGAAAACUGGAC |
| hsa-miR-145-5p mimics nc | F:UCACAACCUCCUAGAAAGAGUAGA<br>R:UCUACUCUUUCUAGGAGGUUGUGA |
| hsa-miR-145-5p inhibitor | AGGGAUUCCUGGGAAAACUGGAC |
| hsa-miR-145-5p inhibitor nc | UCUACUCUUUCUAGGAGGUUGUGA |

**Table S5.** Sequences of UCA1-WT/MUT and SOCS7-WT/MUT

| Plasmid | Sequences |
|---|---|
| UCA1-WT | TTCGGGTAACTCTTACGGTGGAGGATTCCCAGCCATATGAAGA<br>CACCCTAGCTGGACGATCAGTCCTTGTCAAAAGTCTGACCCC<br>TCAAACTCTACAGCCTCAATGGACCAGACCCTACC |
| UCA1-MUT | TTCGGGTAACTCTTACGGTGGAGGATTCCCAGCCATATGAAGA<br>CACCCTAGGATTCGGATCAGTCCTTGTCAAAAGTCTGACCCCT<br>CAAACTCTACAGCCTCAATGGACCAGACCCTACC |
| SOCS7-WT | CTCACACCCACCCCTGGGCATCTTAGGACTGGAGGGGCTCCT<br>TGGAAAACTGGAAGAAGTCTCAACACTGTTTCTTTTTCAGAA<br>GTTTTGTTTTTGATATTTATATTACT TGGTATGGAA |
| SOCS7-MUT | CTCACACCCACCCCTGGGCATCTTAGGACTGGAGGGGCTCCT<br>TGGAAACTGATCAGAAGTCTCAACACTGTTTCTTTTTCAGAA<br>GTTTTGTTTTTGATATTTATATTACTTGGTATGGAA |
